# Non-Hodgkin's lymphoma in a woman with adult-onset Still's disease: a case report

**DOI:** 10.1186/1752-1947-2-73

**Published:** 2008-03-06

**Authors:** Zaher K Otrock, Hassan A Hatoum, Imad W Uthman, Ali T Taher, Shahrazad Saab, Ali I Shamseddine

**Affiliations:** 1 Department of Pathology and Laboratory, American University of Beirut Medical Center, Beirut, Lebanon; 2 Department of Internal Medicine, American University of Beirut Medical Center, Beirut, Lebanon

## Abstract

**Introduction:**

Adult onset Still's disease is a chronic multisystemic inflammatory disorder characterized by high spiking fever, polyarthralgia and rash. Lymphadenopathy is a prominent feature of adult onset Still's disease and is seen in about 65% of patients. Searching the medical literature using the MEDLINE database from January 1966 through November 2007 we could only find two reported cases of adult onset Still's disease that had progressed to lymphoma.

**Case presentation:**

We describe a woman who was diagnosed with adult onset Still's disease and developed lymphoma 10 months after the onset of her symptoms. She initially presented with fever and arthritis of the knees, ankles and shoulders, along with a nonpruritic skin rash, myalgia and weight loss. On physical examination she was found to have several enlarged anterior cervical lymph nodes and left posterior auricular lymph nodes all of which were non-tender, immobile and rubbery. Excisional biopsy of the cervical lymph nodes was negative for malignancy. Bone marrow biopsy was also negative for malignancy. She was treated with prednisone. She remained in good health until she presented 10 months later with low back pain, dyspnea and weight loss. Work up revealed malignant lymphoma. She was treated with chemotherapy and was doing well until she presented with abdominal pain. Work up revealed a cirrhotic liver and ascites. She then passed away from hepatorenal syndrome 13 years after the diagnosis of lymphoma. To our knowledge, this is the third reported case of such an occurrence.

**Conclusion:**

Although the association between adult onset Still's disease and lymphoma has been rarely reported, careful screening for this malignancy in patients suspected to have adult onset Still's disease is warranted.

## Introduction

Adult onset Still's disease (AOSD) is a chronic multisystemic inflammatory disorder of unknown origin characterized by a high spiking fever, polyarthralgia, a salmon pink evanescent rash, and hepatosplenomegaly [1]. Notable laboratory features of the disease are increased serum levels of C-reactive protein (CRP), leukocytosis, liver dysfunction, negative results for both rheumatoid factor and antinuclear antibodies, and an increased incidence of hyperferritinemia [2,3]. Lymphadenopathy is a prominent feature of AOSD seen in about 65% of patients [4] and necessitating in most instances a biopsy to rule out lymphoma.

We describe a woman who was diagnosed with AOSD and developed non-hodgkin's lymphoma (NHL) 10 months after the onset of her symptoms. To our knowledge, this is the third reported case of such an occurrence.

## Case presentation

A 32-year-old woman presented in 1991 with a 2-month history of fever reaching 39.5°C and associated with arthritis in the knees, ankles and shoulders, a nonpruritic skin rash, myalgia and weight loss. Her laboratory studies, including liver function tests, were within the normal ranges except that she had an elevated erythrocyte sedimentation rate (ESR) of 110 mm/hr and lactate dehydrogenase (LDH) of 1975 IU/L (Normal Range: 200–480). The rheumatoid factor and antinuclear antibodies were negative. Blood cultures were also negative. On physical examination she was found to have several enlarged right anterior cervical lymph nodes (2 × 3 cm in size) and left posterior auricular lymph nodes (0.5 × 1 cm in size) all of which were non-tender, immobile and rubbery. In addition, she had swelling in both knee joints and ankle joints. Papular skin lesions on the neck and upper abdomen were evident. No hepatosplenomegaly was detected. A chest radiograph revealed pleural effusion in the left lower lung lobe. Computed tomography (CT) scan of the abdomen and pelvis was normal. Excisional biopsy of cervical lymph nodes done at another hospital and reviewed by our pathologist was negative for malignancy. Bone marrow biopsy was negative for malignancy. Celiac angiogram to rule out vasculitis was negative. A diagnosis of AOSD was made based on the Yamaguchi criteria [5]. She had 2 major criteria: arthritis and fever, and 3 minor criteria: lymphadenopathy, elevated LDH and negative rheumatoid factor and antinuclear antibodies. The patient was started on steroid therapy.

**Figure 1:**
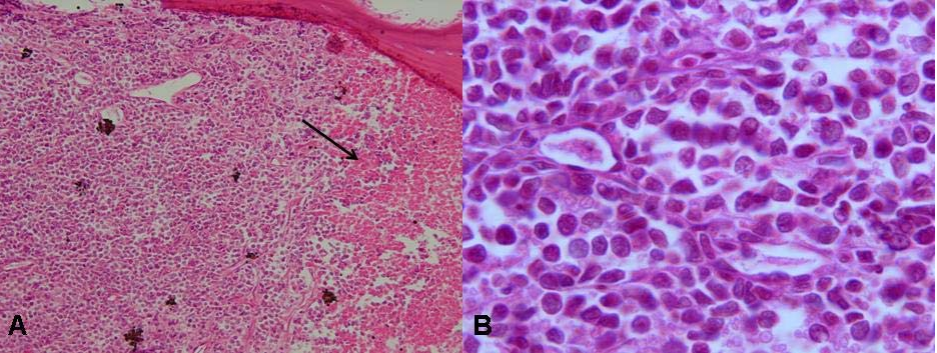
A – The bone marrow cellularity was 95% with large areas of necrosis comprising approximately 10% of the bone marrow core (arrow). B – Diffuse infiltration of bone marrow with a monomorphic population of large atypical lymphocytic cells consistent with large cell lymphoma.

**Figure 2:**
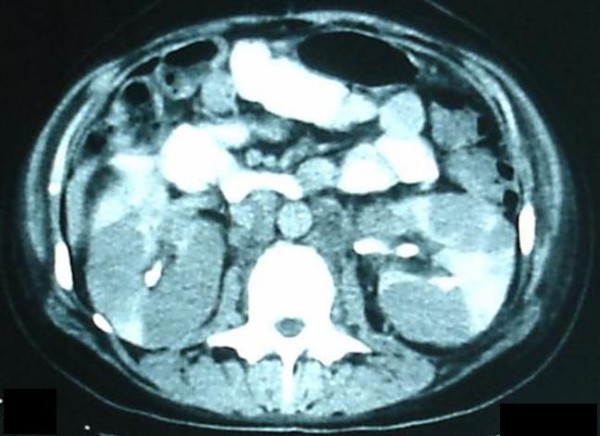
CT scan of the abdomen showing enlarged retroperitoneal lymph nodes and infiltration of both kidneys with hypodense masses compatible with lymphomatous involvement.

She remained in good health, maintained on low dose prednisone (5 mg/day), until she presented 10 months later with low back pain and dyspnea, associated with weight loss of 5 kg over 2 months. Blood studies showed hemoglobin of 7 gm/dl and hematocrit of 22 %. She was transfused with packed red blood cells. Bone marrow biopsy showed infiltration with malignant lymphoma of large cell type with extensive necrosis (Figure 1). CT scan of the abdomen and pelvis showed enlarged retroperitoneal lymph nodes and infiltration of the kidneys with hypodense masses compatible with lymphomatous involvement (Figure 2). Bone scan was compatible with bone disease. She received one cycle of adriamycin and Ara-C followed by four cycles of ProMACE and CytaBOM chemotherapy with adequate response initially. One month later, she started having high grade fever, headache and generalized aches. Re-evaluation revealed CNS relapse with CSF involvement with malignant lymphoma cells, and without evidence of systemic disease. She was treated with intrathecal methotrexate, intermediate dose Ara-C, high dose methotrexate and whole brain irradiation. She achieved complete remission and was doing well till May 2003 when she presented with abdominal pain. Work up revealed a cirrhotic liver and ascites. She passed away from hepatorenal syndrome 13 years after the diagnosis of lymphoma.

## Conclusion

We described a case of a woman with AOSD who was diagnosed with lymphoma 10 months after the onset of symptoms. The patient satisfied the criteria of Yamaguchi et al for AOSD [5]. In this case, there is a possibility that the lymphoma had latently existed from the beginning of the clinical course. However, 10 months had elapsed after the onset of Still's disease before the development of symptoms of lymphoma and a previous lymph node biopsy had been negative for lymphoma.

AOSD is commonly considered in the differential diagnosis of fever of unknown origin, especially if associated with multiple organ involvement [4,6]. In the absence of specific clinical, laboratory and histological features for AOSD, the exclusion of infections, malignancies and other rheumatologic diseases is crucial.

Lymphadenopathy commonly occurs in AOSD [4]. Although most of the histopathologic studies performed have shown non-diagnostic reactive hyperplasia in AOSD, histological patterns simulating malignant lymphoma have been reported. Besides necrotizing lymphadenitis [7], some authors have described a distinctive, intense paracortical hyperplasia characterized by an expansion of immunoblastic cells and prominent arborizing vessels [8,9]. It has been pointed out that the intensity of the process, along with the apparent nodal architecture effacement and the atypical proliferating cells, may suggest an erroneous diagnosis of malignancy. Sometimes it may be difficult to differentiate AOSD from malignant hematologic disorders [10]. In addition to clinical features, histopathological features of lymph node biopsy may also mimic lymphoma [11,12].

We searched the medical literature using the MEDLINE database from January 1966 through November 2007. Only two cases of AOSD that progressed to lymphoma were reported. In 1993, Trotta et al reported a case of AOSD associated with an immunoblastic malignant lymphoma [13] and in 2000 Sono et al reported a case that progressed to diffuse large B-cell lymphoma [14]. (See Table 1) To our knowledge, this report is the third reported case of such an association. Our patient was the youngest among the three reported cases and her lymphoma was diagnosed 10 months after the onset of AOSD. Although the association between AOSD and malignant lymphoma has been rarely reported, careful screening for this malignancy in patients suspected to have AOSD is very important.

**Table 1:** Summary of reported cases with AOSD who developed lymphoma.

Reference	Age at diagnosis of AOSD (years)	Clinical presentation	Lymphadenopathy	Time elapsed from symptoms to lymphoma diagnosis (months)	Treatment	Survival
Trotta et al	52	Fever, arthritis	Absent but with hepatosplenomegaly	21	unknown	unknown
Sono et al	50	Fever, arthritis, myalgia	Absent	18	Chemotherapy	3 month follow-up
Our case	32	Fever, arthralgia	Present	10	Chemo-and radiotherapy	13 years

## Abbreviations

AOSD: Adult Onset Still's Disease; CRP: C-reactive protein; NHL: non-hodgkin's lymphoma; ESR: erythrocyte sedimentation rate; LDH: lactate dehydrogenase; CT: Computed tomography

## Competing interests

The author(s) declare that they have no competing interests.

## Authors' contributions

All authors were involved in writing and/or reviewing of this manuscript. All authors approved the final version of the manuscript.

## Consent section

Written informed consent was obtained from the patient's family for publication of this case report and accompanying images. A copy of the written consent is available for review by the Editor-in-Chief of this journal.
